# Tricetin Reduces Inflammation and Acinar Cell Injury in Cerulein-Induced Acute Pancreatitis: The Role of Oxidative Stress-Induced DNA Damage Signaling

**DOI:** 10.3390/biomedicines10061371

**Published:** 2022-06-10

**Authors:** Máté Nagy-Pénzes, Zoltán Hajnády, Zsolt Regdon, Máté Á. Demény, Katalin Kovács, Tarek El-Hamoly, József Maléth, Péter Hegyi, Csaba Hegedűs, László Virág

**Affiliations:** 1 Department of Medical Chemistry, Faculty of Medicine, University of Debrecen, 4032 Debrecen, Hungary; nagy.mate@med.unideb.hu (M.N.-P.); hajnady.zoltan@med.unideb.hu (Z.H.); regdike1988@gmail.com (Z.R.); kovacs.katalin@med.unideb.hu (K.K.); tahamoly@hotmail.com (T.E.-H.); hcsaba@med.unideb.hu (C.H.); 2 Doctoral School of Molecular Medicine, University of Debrecen, 4032 Debrecen, Hungary; 3 MTA-DE Cell Biology and Signaling Research Group, 4032 Debrecen, Hungary; demenym@med.unideb.hu; 4 Drug Radiation Research Department, National Centre for Radiation Research and Technology, Egyptian Atomic Energy Authority, Cairo 11787, Egypt; 5 First Department of Medicine, University of Szeged, 6720 Szeged, Hungary; maleth.jozsef@med.u-szeged.hu (J.M.); hegyi2009@gmail.com (P.H.); 6 HAS-USZ Momentum Epithelial Cell Signalling and Secretion Research Group, 6720 Szeged, Hungary; 7 Department of Public Health, University of Szeged, 6720 Szeged, Hungary; 8 Institute for Translational Medicine, János Szentágothai Research Centre, University of Pécs Medical School, 7624 Pécs, Hungary; 9 Division of Pancreatic Diseases, Heart and Vascular Center, Semmelweis University, 1122 Budapest, Hungary

**Keywords:** acute pancreatitis, tricetin, inflammation, cell death, necrosis, oxidative stress

## Abstract

Acute pancreatitis (AP) poses a worldwide challenge due to the growing incidence and its potentially life-threatening course and complications. Specific targeted therapies are not available, prompting the identification of new pathways and novel therapeutic approaches. Flavonoids comprise several groups of biologically active compounds with wide-ranging effects. The flavone compound, tricetin (TCT), has not yet been investigated in detail but sporadic reports indicate diverse biological activities. In the current study, we evaluated the potential protective effects of TCT in AP. TCT (30 μM) protected isolated primary murine acinar cells from the cytotoxic effects of cerulein, a cholecystokinin analog peptide. The protective effects of TCT were observed in a general viability assay (calcein ester hydrolysis), in an apoptosis assay (caspase activity), and in necrosis assays (propidium iodide uptake and lactate dehydrogenase release). The effects of TCT were not related to its potential antioxidant effects, as TCT did not protect against H_2_O_2_-induced acinar cell death despite possessing radical scavenging activity. Cerulein-induced expression of IL1β, IL6, and matrix metalloproteinase 2 and activation of nuclear factor-κB (NFκB) were reduced by 30 μM TCT. In vivo experiments confirmed the protective effect of TCT in a mouse model of cerulein-induced AP. TCT suppressed edema formation and apoptosis in the pancreas and reduced lipase and amylase levels in the serum. Moreover, TCT inhibited interleukin-1β (IL1β), interleukin-6 (IL6), and tumor necrosis factor-α (TNFα) expression in the pancreas and reduced the activation of the oxidative DNA damage sensor enzyme poly(ADP-ribose) polymerase-1 (PARP-1). Our data indicate that TCT can be a potential treatment option for AP.

## 1. Introduction

The pancreas functions as an exocrine and endocrine organ [1]. The secretion of pancreatic juice containing digestive enzymes (e.g., chymotrypsin, trypsin, lipase, amylase, etc.), protease inhibitors, and bicarbonate into the intestine is termed the exocrine function of the pancreas [2,3]. The endocrine function includes the production of insulin, glucagon, somatostatin, ghrelin, and amylin by cells of the islets of Langerhans [4,5]. Pancreatitis is an autolytic process initiated in the exocrine pancreas. Autoactivation of zymogenic digestive enzymes is typically triggered by binge alcohol consumption or the formation of bile stones blocking the terminal section of the common bile duct (papilla Vateri) through which both the pancreatic juice and the bile are secreted [2]. Hypertriglyceridemia, infections, toxins, drugs, endoscopic retrograde cholangiopancreatography, and surgery are also linked to acute pancreatitis (AP) and about 10% of AP cases are classified as idiopathic [6,7]. The incidence of AP varies between 13 and 45 cases/100,000 people in developing countries [8] and the incidence of AP is increasing in many countries. The severity of AP ranges from mild, spontaneously healing forms to life-threatening severe cases [9].

Although drugs are generally overused in AP [10,11], treatment options for AP are limited and include two-phases of fluid resuscitation [12] and early enteral feeding [13]. Targeted therapies are not available despite extensive efforts to identify molecular targets for AP treatment.

Several novel treatments were identified in preclinical studies; however, translation of these efforts into the clinic was mostly unsuccessful. Therapies that work in preclinical models of AP include, but are not limited to, nonsteroidal anti-inflammatory drugs (e.g., indomethacin, diclofenac, ketorolac) [14], pentoxifylline [15], calcium channel inhibition with CM4620 [16], poly(ADP-ribose) polymerase (PARP) inhibitors [17,18,19], peptide hormones (e.g., somatostatin, secretin) [20,21], hemin [22], neostigmine [23], and blocking the TNFα receptor with infliximab [24]. Moreover, various dietary supplements and natural products (e.g., glutamine, resveratrol, spilanthol) [25,26,27,28,29] were also effective in preclinical models of AP. Several ongoing clinical trials aim to determine which of these novel therapeutic approaches can be translated to the clinic.

Polyphenols, including compounds of the flavonoid family, have anti-pancreatitis potential [30]. Flavonoids share the 2-phenyl-1,4 benzopyrone structure, known as the flavone backbone. Flavonoids from most subgroups have been evaluated for potential protective effects in AP. For example, genistein (isoflavone) [31], quercetin (flavonol) [32], naringenin (flavanone) [33], apigenin (flavone) [34], dihydromyricetin (flavanonol) [35], luteolin (flavone) [36], and fisetin (flavonol) [37] either suppressed inflammation, prevented acinar cell damage, or both [38,39]. Tricetin (TCT) (Supplementary Figure S1B) is a flavone compound with likely or proven bioactivity in models of neurotoxicity, Parkinson’s disease [40], diabetes [41,42], and bacterial infections [43]. Most studies focus on the potential anticancer and anti-metastatic effects of TCT [44,45,46]. However, the effects of TCT in AP have not been investigated. Data reporting the anti-inflammatory effects of TCT in other types of inflammations are also scarce [47,48,49]. Therefore, the aim of this study was to determine if TCT has a protective effect in isolated acinar cells and in an animal model of AP. Our data show that TCT is an effective cytoprotective and anti-pancreatitis agent in a cerulein-induced acinar cell injury model.

## 2. Materials and Methods

### 2.1. Isolation and Culture of Pancreatic Acinar Cells

Pancreatic acinar cells were isolated from male and female C57BL/6j mice and prepared by collagenase digestion as previously described [50]. The cells were cultured in a 5% CO_2_ incubator at 37 °C. The cells were grown in endotoxin-free high glucose Dulbecco’s modified Eagle’s medium (Sigma-Aldrich, St. Louis, MO, USA) supplemented with 2.5% fetal bovine serum, 1% penicillin-streptomycin mixture, 0.25 mg/mL of trypsin inhibitor (Sigma-Aldrich, St. Louis, MO, USA), and 25 ng/mL of recombinant human epidermal growth factor (Sigma-Aldrich, St. Louis, MO, USA).

### 2.2. Cell Treatments

Cells were seeded in 96-well tissue culture plates for viability and cytotoxicity assays, 24-well tissue culture plates for immunofluorescence (IF), and 6-well tissue culture plates for reverse transcription quantitative real-time polymerase chain reaction (RT-qPCR) assays, Western blotting, and NFκB p65 binding assays. Surfaces of the plates were coated with laminin (Sigma-Aldrich, St. Louis, MO, USA) 1 h before seeding. Laminin was discarded and plates were washed with PBS and dried. One hour of pretreatment with various concentrations of TCT (Extrasynthese, Genay, France) was followed by treatment with 100 nM cerulein (Sigma-Aldrich, St. Louis, MO, USA) for 24 h. Cerulein and TCT were dissolved in dimethyl sulfoxide (DMSO) (stock solution). DMSO concentrations were below 0.1% (*v*/*v*) in cell cultures and did not have any effects on the parameters measured.

### 2.3. Radical Scavenging Assay

The ABTS (2,2′-azinobis-3-ethylbenzothiazoline-6-sulfonic acid) assay was used to determine the radical scavenging (antioxidant) effect of TCT using vitamin C (Sigma-Aldrich, St. Louis, MO, USA) as a positive control. ABTS is a chromogenic free radical generated overnight by combining it with potassium persulfate (7.4 mM ABTS (Merck Millipore, Burlington, MA, USA) + 10% 24.5 mM K-persulfate). Antioxidant properties of the compounds were determined by the decolorization of the intense green ABTS radical solution. The ABTS solution was prepared as described [51]. The ABTS solution was diluted with 50 mM Gly-HCl buffer to reach a concentration where the absorbance of the solution was A = 1 (1 cm light path at 405 nm) in a Spark photometer (Tecan Spark, Männedorf, Switzerland). Three-fold serial dilutions of TCT and vitamin C were pipetted into 96-well plates containing the assay solution and after 30 min incubation time, absorbance was measured at 405 nm.

### 2.4. Cell Viability (Calcein-AM Assay)

A calcein-AM (Sigma-Aldrich, St. Louis, MO, USA) stock solution (4 mM in DMSO) was diluted in medium and was added to the cells cultured in 96-well plates (1 µM final concentration). Calcein-AM was also added to blank wells containing no cells but cell-free medium. After 40 min incubation at 37 °C, fluorescence intensity of the wells containing medium with or without cells was determined at 485/535 nm in a Spark plate reader. Viability was calculated and expressed as a percentage of the control cells.

### 2.5. Lactate Dehydrogenase (LDH) Assay

LDH activity was determined with a commercially available Cytoscan LDH-assay kit purchased from G-Biosciences (St. Louis, MO, USA) following the kit’s protocol with slight modifications as follows. Briefly, 50 μL of culture medium was transferred from the cells to 96-well half-area microplates and 50 µL of LDH reagent mixture was added to each well. Supernatants of lysed control cells (prepared by the addition of 10% Triton-X in PBS for 10 min) were used as a positive control (100% LDH activity). After 20 min incubation at room temperature, the absorbance was measured at 490 nm in a Spark microplate reader. Cytotoxicity was calculated with the following equation:

Relative LDH release (%) = 100 × (OD_sample_ − OD_control_)/(OD_lysate_ − OD_control_)

### 2.6. Propidium Iodide (PI) Uptake

Acinar cells were isolated and seeded into CellCarrier Ultra 96-well microplates (Perkin Elmer, Waltham, MA, USA). Cells were treated with TCT and cerulein or hydrogen peroxide for 24 h. Cells were then stained with propidium iodide (2.5 μg/mL) (Sigma-Aldrich, St. Louis, MO, USA) and Hoechst342 (5 μg/mL) (Sigma-Aldrich, St. Louis, MO, USA) for 15 min. Images were analyzed as previously reported [52] with an Opera Phenix High-Content Analysis system (Perkin Elmer, Waltham, MA, USA) using a 40× air objective and appropriate laser and filter settings in sequential mode to avoid overlapping of the emission spectra.

### 2.7. Caspase Activity Assay

Acinar cells were isolated and seeded into CellCarrier Ultra 96-well microplates (Perkin Elmer, Waltham, MA, USA). Cells were pretreated for 1 h with TCT (30 μM) and then treated with cerulein (100 nM). CellEvent Caspase-3/7 Green reagent (5 μM; Thermo Fisher Scientific, Waltham, MA, USA) was added and fluorescence of the CellEvent reagent was detected every 3 h for 18 h with an Opera Phenix High-Content Analysis system (Perkin Elmer, Waltham, MA, USA) using a 40× air objective and appropriate laser and filter settings. Cells were identified in bright field mode.

### 2.8. RNA Extraction, Reverse Transcription, and qPCR Analysis

Acinar cells were isolated and seeded into 6-well plates. After treatment (or after collection of pancreas tissues from mice), total RNA was isolated from cells/tissues with TRI reagent (Molecular Research Center, Cincinnati, OH, USA) and RNA purity was checked by measuring the A260/280 ratio. RT-qPCR was performed as described previously [52]. Gene expression levels were normalized to the harmonic mean of 36B4 and RPS26, as reference housekeeping genes. Primers used for RT-qPCR are summarized in Table 1.

### 2.9. NFκB Assay

To investigate the effect of TCT on nuclear translocation and consensus sequence binding of the NFκB p65 subunit, an NFκB p65 Transcription Factor Assay Kit (Abcam, Cambridge, UK) was used according to the manufacturer’s instructions. The cells were pretreated with 30 μM TCT, or medium for 1 h and were then treated with a combination of 20 ng/mL murine interferon-γ (mIFNγ) (Sigma-Aldrich, St. Louis, MO, USA) and 1 μg/mL lipopolysaccharide (LPS) (Sigma-Aldrich, St. Louis, MO, USA) or with 1 μM phorbol 12-myristate 13-acetate PMA (Sigma-Aldrich, St. Louis, MO, USA) for 1 h. Nuclear fractions were extracted from the cells using the protocol supplied with the kit. To investigate the direct effect of TCT on NFκB DNA binding, TCT (30 μM) was added to some nuclear extract samples 1 h before the binding assay. Absorbance was measured at 450 nm and DNA binding capacity of the p65 subunit was expressed as a percentage of the control.

### 2.10. PARP Inhibition Assay

To determine the PARP inhibitory effect of TCT, a PARP Activation Kit (Trevigen, Gaithersburg, Maryland, USA) was used according to the manufacturer’s instructions; 3-aminobenzamide (3-AB) was used as a positive PARP inhibitor control. The concentrations of TCT and 3-AB were 10 mM, 1 mM, 300 μM, 100 μM, 30 μM, 10 μM, 3 μM, 1 μM, 0.3 μM, 0.1 μM, and 0.1 nM.

### 2.11. Immunofluorescent (IF) Staining of Acinar Cells

Acinar cells were isolated and seeded onto 13 mm coverslips (Thermo Fisher Scientific, Waltham, MA, USA). The cells were treated with 250 µM hydrogen peroxide (7.5 min) with or without 10–30 µM TCT pretreatment (1 h). Coverslips were incubated in methanol for 20 min at −20 °C, washed with phosphate-buffered saline (PBS), and lysed with 10% Triton-X in PBS. After blocking in 1% bovine serum albumin (BSA) in PBS at room temperature, cells were incubated with a mouse monoclonal poly(ADP-ribose) (PAR) specific antibody (purified in-house from the supernatant of the 10H hybridoma; isotype IgG3) overnight at 4 °C. On the following day, cells were incubated with a goat anti-mouse Alexa633-conjugated secondary antibody (Thermo Fisher Scientific, Waltham, MA, USA) (1:1000) and 4′,6-diamidino-2-phenylindole (DAPI) (Sigma-Aldrich, St. Louis, MO, USA) (1:2000) at room temperature. The coverslips were viewed with an SP8 Leica confocal microscope. The intensity of the PAR signal was analyzed with ImageJ software.

### 2.12. Western Blot Analysis

Acinar cells were isolated and seeded into 6-well tissue culture plates. The cells were treated with 250 µM hydrogen peroxide for 7.5 min with or without 10 and 30 µM TCT pretreatment (1 h). Total protein lysates were separated on 8% SDS-PAGE gels at 100 V for 90 min. Proteins were transferred to nitrocellulose membranes. Membranes were blocked with 5% BSA in phosphate buffered saline with Tween^®^ 20 (PBST) for 1 h at room temperature. Membranes were incubated with antibodies against PAR (clone 10H; purified in-house) overnight at 4 °C. After washing with Tris buffered saline with Tween^®^ 20 (TBST), membranes were incubated with peroxidase-conjugated anti-mouse IgG (1:3000; Cell Signaling, Danvers, MA, USA) and anti-β-actin (1:20,000; Santa Cruz Biotechnology, Santa Cruz, CA, USA) antibodies for 2 h at room temperature. Membranes were then washed with PBST and incubated with WestFemto chemiluminescent reagent (Thermo Fisher Scientific, Waltham, MA, USA). Luminescence was detected with a Chemidoc Touch gel documentation system (BioRad Laboratories, Hercules, CA, USA). Signal intensities were quantified using Image Lab software (BioRad). The blot area used for signal quantification was selected to cover the PARylated PARP1 region (ca. 100–180 kDa) and actin signals were used for normalization.

### 2.13. Acute Pancreatitis Model

Animal experiments were approved by the institutional animal welfare committee of University of Debrecen protocol number: 25/2017/DEMÁB. Mice (C57BL/6j, 8–10 weeks old) were bred and maintained at 21–23 °C, 30–60% relative humidity, and a 12 h light/dark cycle. Mice were housed in Eurostandard Type II cages and were fed ad libitum with VRF1 (P) food and water. Acute pancreatitis was induced as previously described [53] with modification as follows. Male mice (*n* = 18) were randomized into three groups: control, cerulein, and cerulein + TCT. Mice in the control group were given saline only. In the cerulein-treated group, AP was induced by eight intraperitoneal injections of cerulein (50 μg/kg BW) at hourly intervals. In the cerulein + TCT group, mice received two intraperitoneal injections of TCT (10 mg/kg BW) 12 h and 1 h before the first cerulein injection.

We also tested the in vivo efficacy of TCT in a posttreatment setting. Control (*n* = 5), cerulein-treated (n = 6), and cerulein + TCT-treated mice (*n* = 6) received two i.p. injections of TCT (30 mg/kg BW) between the fourth and fifth cerulein injections and 10 mg/kg BW of TCT after the last cerulein injection. We chose TCT doses mainly based on literature data on the effects of flavonoids in the mouse model of AP. The flavonol compound fisetin [37] and the prenylated flavonol glycoside icariin [54] were both administered i.p. and their doses were the same as ours (10 mg/kg). The flavonoid most closely related to TCT luteolin was used in 25–50–100 mg/kg doses in a study [36]. Since a posttreatment is always less effective than a pretreatment, for the posttreatment protocol we chose a higher first dose (30 mg/kg) to quickly reach a potentially therapeutic serum concentration and a low second dose (10 mg/kg) for “maintenance” therapy.

Mice in the control and cerulein groups were given saline (with 0.3% DMSO) instead of TCT. The DMSO contents of the cerulein and TCT solutions were 0.1% and 0.3%, respectively. Mice were sacrificed 10 h after the first cerulein injection in both the pretreatment and posttreatment experiments and blood and pancreas tissues were collected.

### 2.14. Serum α-Amylase and Lipase Determination

Blood samples were taken by cardiac puncture and were centrifuged at 5000× *g* for 10 min at 4 °C. Serum α-amylase and lipase activities were measured (using an enzymatic assay kit from Diagnosticum Zrt., Budapest, Hungary) in a kinetic reaction over 20 min according to the manufacturer’s instructions using a Spark plate reader at 405 nm (α-amylase) and 580 nm (lipase). Absorbance was measured every 15 s to generate a kinetic curve. The slope of the curves was related to the slope of the control sample curve.

### 2.15. Myeloperoxidase Assay

Tissue myeloperoxidase (MPO) activity was measured as an indicator of neutrophil infiltration in the pancreas, as described previously [26] with modifications as follows. Tissue samples were thawed, homogenized in 1 mL of 20 mM phosphate buffer (pH 7.4), and centrifuged at 13,000× *g* for 30 min at 4 °C. The resulting pellet was resuspended in 0.5 mL of 50 mM phosphate buffer (pH 6.0) containing 0.5% hexadecyltrimethylammonium bromide (Sigma-Aldrich, St. Louis, MO, USA). The homogenates were then frozen in liquid nitrogen and thawed on three consecutive occasions before sonication. Total protein concentration was measured with a Direct Detect Spectrometer (Merck Millipore, Burlington, MA, USA). The samples were then centrifuged (13,000× *g* for 30 min at 4 °C), and the supernatants were collected for the MPO assay. Supernatants (100 µL) were mixed with 100 µL substrate solution containing 1.6 mM 3,3′,5,5′-tetramethylbenzidine (Sigma Aldrich, St. Louis, MO, USA) and 1 mM hydrogen peroxide. The mixture was incubated at 37 °C for 90 s, and the reaction was stopped with 200 μL 2 M H_2_SO_4_. The absorbance was measured using a Tecan Spark plate reader at 450 nm and then normalized to total protein content.

### 2.16. Histology

Pancreatic tissues were collected and fixed in 10% formalin for 48 h. Dehydration, embedding, sectioning, and staining with hematoxylin and eosin were performed according to guidelines in [55]. The TUNEL (terminal deoxynucleotidyl transferase dUTP nick end labeling) assay was performed with a TUNEL Assay Kit—HRP—DAB (Abcam, Cambridge, UK) according to the manufacturer’s instructions.

Staining was scored by a pathologist blinded to the experimental design and identity of the samples. A scoring scheme from 0 to 3 for edema, leukocyte infiltration, necrosis, and TUNEL assay was used [56], as presented in Table 2.

### 2.17. Immunofluorescent (IF) Detection of PAR Polymers in Tissue Sections

Poly(ADP-ribose) (PAR) polymer was detected by immunofluorescence, as described previously [57]. Sections (5 μm) of pancreatic tissue were blocked with 1% BSA in PBS for 1 h at room temperature and then incubated with mouse monoclonal poly(ADP ribose) antibody (purified in-house) at 3 μg/mL concentration overnight at 4 °C. The tissues were then incubated with Alexa633-conjugated goat anti-mouse IgG secondary antibody (Thermo Fisher Scientific, Waltham, MA, USA) (1:1000), and DAPI (Sigma-Aldrich, St. Louis, MO, USA) (1:2000) at room temperature. Sections were viewed with a Leica Sp8 Confocal Microscope (Leica, Wetzlar, Germany). The PAR signal was analyzed using ImageJ software. PARylation rates were calculated and expressed as intensity values.

### 2.18. Statistical Analysis

In vitro experiments were repeated at least three times and all values are expressed as mean ± SD. The Kolmogorov-Smirnov test was used to check if continuous variable data displayed normal distribution. All data showed normal distribution. One- or two-way ANOVA followed by a Tukey post hoc test was used for analysis. Discrete variables (scores of micrographs) were analyzed using Fisher’s exact test. The value of *p* < 0.05 was considered significant.

## 3. Results

### 3.1. Tricetin Is Nontoxic to Acinar Cells

Isolated acinar cells are a good cell model for studying the exocrine pancreas. We isolated primary acinar cells from mice and tested the toxicity of TCT. Cell viability was evaluated with calcein assay and plasma membrane injury was assessed with LDH release assay using hydrogen peroxide (1 mM) as a positive control. We found that TCT was non-toxic to primary acinar cells up to a concentration of 30 μM (Supplementary Figure S1). At three times higher concentration (100 μM), TCT had a small but significant toxic effect on acinar cells. This could be observed in both the calcein and LDH release assays (Supplementary Figure S1). Therefore, we chose 30 μM TCT for the subsequent cell-based experiments.

### 3.2. Tricetin Protects Isolated Acinar Cells from Cerulein-Induced but Not from Hydrogen Peroxide-Induced Cell Death

**Figure 1:**
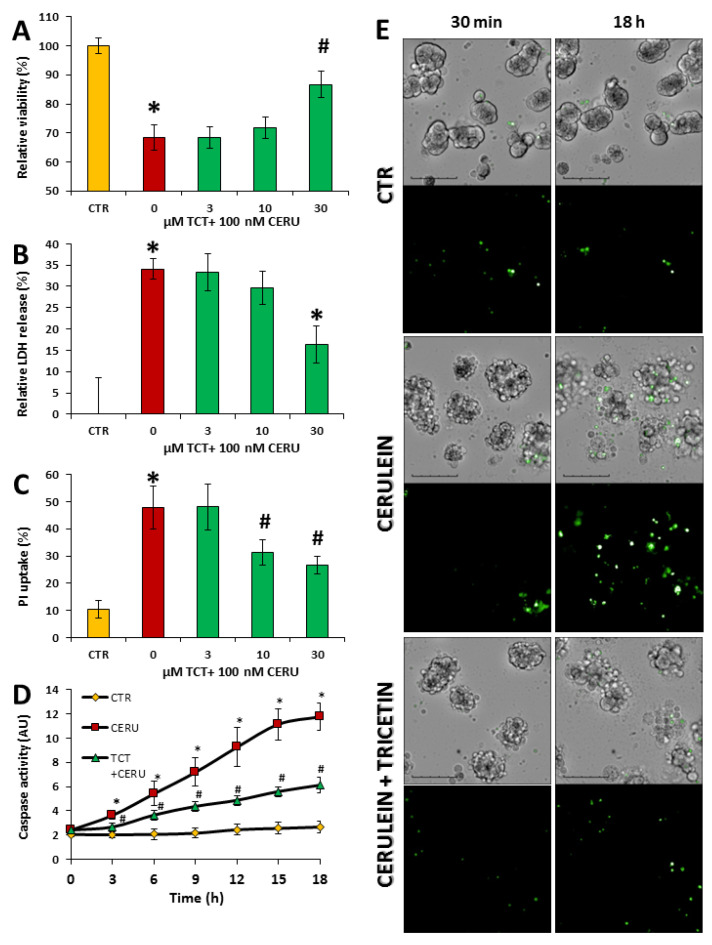
Tricetin protects isolated acinar cells from cerulein-induced cell death. Primary acinar cells were pretreated with 0–30 μM tricetin (TCT) for 1 h and then treated with 100 nM cerulein for 24 h. Viability was determined with calcein assays (**A**), and necrotic cell death was measured by LDH release (**B**) and propidium iodide (PI) uptake assays (**C**). Primary acinar cells were pretreated with 30 μM tricetin (TCT) or medium for 1 h and then treated with 100 nM cerulein. Caspase activity was detected every 3 h for 18 h as described in the Materials and Methods section (**D**). Representative images of caspase activity detection at the first and the last time points are shown (**E**). (Scale bar is 100 μm) Error bars represent the SD of three independent measurements. Statistical analysis was performed with one-way ANOVA test followed by Tukey’s post hoc test. Stars (*) indicate significant (*p* < 0.05) difference between cerulein-treated cells versus control. Hashtags (#) indicate significant (*p* < 0.05) cytoprotection by TCT versus the cerulein-treated group.

The cholecystokinin analog peptide, cerulein, is used extensively to model overstimulation-induced acinar cell injury both in cell-based experiments and in vivo. At 100 nM concentration, cerulein decreased cell viability of primary acinar cells, as indicated by the calcein assay (Figure 1A).

This effect was also observed in the LDH release assay (Figure 1B). Pretreatment of the cells with 30 μM TCT significantly protected acinar cells from cerulein-induced injury in both assays (Figure 1A,B). Necrotic cell death and cell membrane injury are critical features of AP; thus, we also examined the effects of TCT on propidium iodide (PI) uptake. The effects of cerulein and TCT on PI uptake were consistent with the results of both the LDH release and the calcein assays (Figure 1C). Apoptotic cell death could also be observed in the cerulein-treated cells as indicated by increased caspase activity (Figure 1D,E). Pretreatment of cells with 30 μM TCT significantly reduced caspase activation (Figure 1D). Representative fluorescent images also illustrate reduced caspase activation in TCT-treated acinar cells (Figure 1E).

TCT displayed radical scavenging activity (ABTS radical scavenging assay) similar to that of vitamin C (Supplementary Figure S2A). Therefore, we tested whether this effect translates to cytoprotection from H_2_O_2_-induced cell injury. H_2_O_2_ caused a concentration-dependent cytotoxicity (Supplementary Figure S2B–D) but TCT had no effect on H_2_O_2_-induced toxicity. Collectively, these results indicate that TCT interferes with the cerulein-induced acinar cell death pathways (Supplementary Figure S2B–D).

### 3.3. Cerulein-Induced Expression of Interleukin-1β (IL-1β), Interleukin-6 (IL6), and Matrix Metalloproteinase-2 (MMP2) Was Attenuated by Tricetin in Primary Pancreatic Acinar Cells

**Figure 2:**
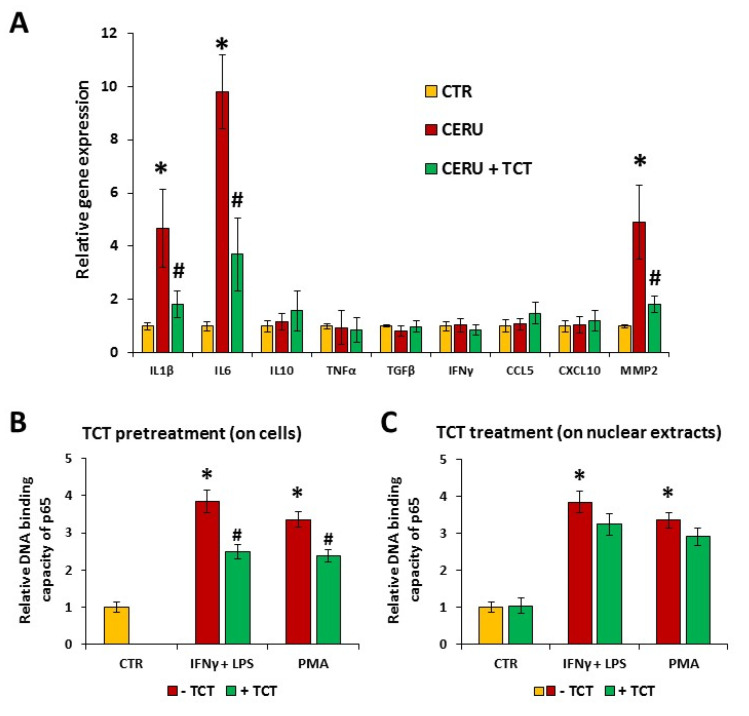
Tricetin reduces the expression of IL1β, IL6, and MMP2 genes, and inhibits NFκB activation in primary pancreatic acinar cells. Primary acinar cells were pretreated with 30 µM tricetin (TCT) or medium for one hour followed by 100 nM cerulein treatment. After 24 h, mRNA levels were measured with RT-qPCR. Error bars represent the SD of five (or three in the cases of CCL5 and CXCL10) independent experiments. Stars (*) indicate significant (*p* < 0.05) differences between cerulein-treated cells versus control. Hashtags (#) indicate significantly (*p* < 0.05) reduced gene expression by TCT versus the cerulein-treated group (**A**). Primary acinar cells were pretreated with 30 µM TCT or medium for one hour followed by 20 ng/mL mIFNγ + 1 μg/mL LPS, or 1 μM PMA treatment for one hour. Nuclear fractions were extracted from the cells and were used in NFκB p65 binding assays as described in the Materials and Methods section (**B**). Part of the nuclear fractions were treated with 30 µM TCT to investigate the effect of TCT on the DNA binding of NFκB (**C**). Statistical analysis was performed with one-way (panel (**A**)) or two-way (panels (**B**,**C**)) ANOVA test followed by Tukey’s post hoc test.

Inflammation contributes to the development of AP. However, AP is unique in the sense that the primary event is cell injury and inflammation develops as a consequence of tissue damage. Nonetheless, suppression of injury-associated inflammation has a beneficial effect on the course of AP. Therefore, the effects of TCT on a set of inflammatory mediators were investigated (Figure 2A).

Cerulein treatment induced the expression of IL1β, IL6, and MMP2 mRNAs, whereas TNFα, IFNγ, TGFβ (transforming growth factor-β), IL10, CCL5 (chemokine (C-C motif) ligand 5), and CXCL10 (C-X-C motif chemokine ligand 10) expression was not induced by cerulein. Pretreatment of acinar cells with TCT reduced the cerulein-induced expression of IL1β, IL6, and MMP2 (Figure 2A).

### 3.4. Tricetin Inhibits NFκB Signaling

We hypothesized that the effect of TCT on cytokine and MMP2 expression may be due to interference with NFκB signaling. In response to various inflammatory signals, NFκB is activated, translocates to the nucleus, and mediates the expression of inflammatory mediator genes. The nuclear translocation of NFκB p65 could be observed in acinar cells treated with IFNγ + LPS or PMA (Figure 2B,C). TCT (30 μM, 1 h) pretreatment significantly decreased nuclear translocation of p65 (Figure 2B). However, addition of TCT to the nuclear extracts had no effect on the DNA binding of p65 subunit (Figure 2C).

### 3.5. Tricetin Inhibits PARP1 and Suppresses H_2_O_2_-Induced Poly(ADP-ribosyl)ation (PARylation) in Isolated Acinar Cells

**Figure 3:**
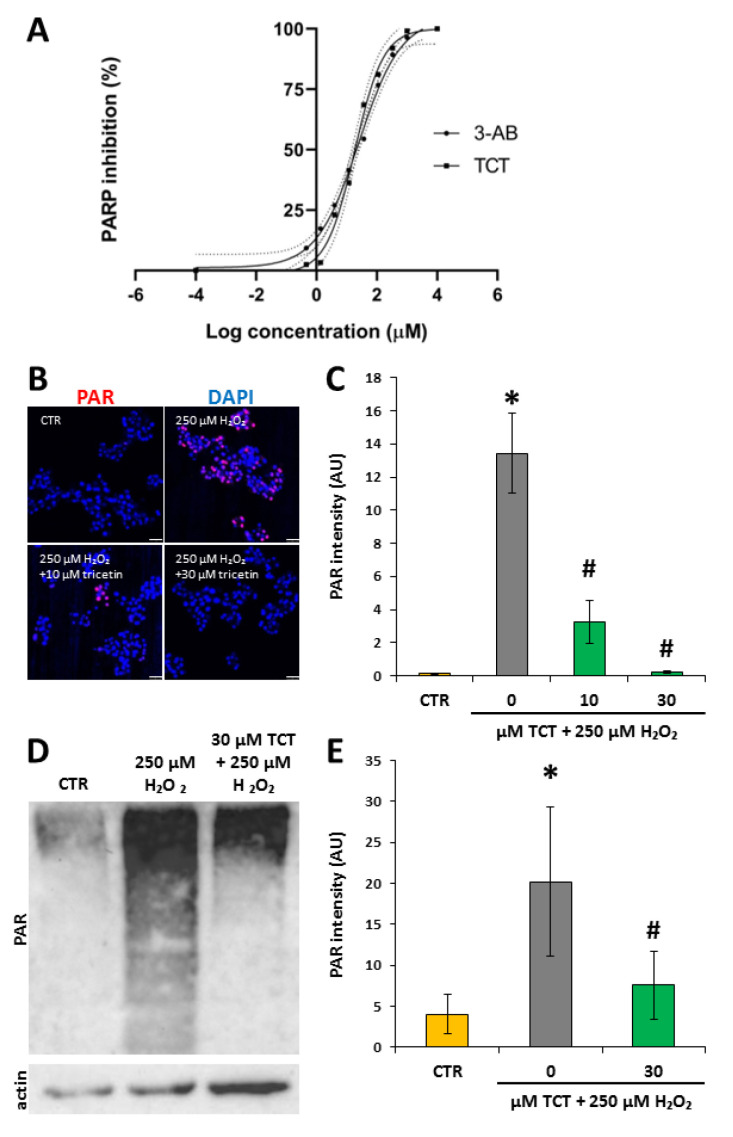
Tricetin inhibits PARP in enzyme activity assays and in isolated acinar cells. Tricetin (TCT) inhibits PARP1, as measured with a PARP activation kit (**A**). Primary acinar cells were pretreated with 10 µM or 30 µM TCT for one hour and then treated with 250 µM hydrogen peroxide for 7.5 min. Immunocytochemical staining of PAR polymer was evaluated with confocal microscopy followed by image analysis with ImageJ software (scale bar 50 μm) (**B**,**C**). PAR polymer, the product of PARP enzymes, was detected in Western blots and signals were quantitated with ImageLab software and were normalized to actin signal intensities (**D**,**E**). Error bars represent the SD of three (IF) or four (WB) independent experiments. Statistical analysis was performed with one-way ANOVA test followed by Tukey’s post hoc test. Stars (*) indicate significant (*p* < 0.05) H_2_O_2_-induced PARylation compared to the control. Hashtags (#) indicate significant (*p* < 0.05) reduction of the PAR signal in TCT-treated samples versus H_2_O_2_-treated cells.

Activation of PARP1 contributes to tissue injury and inflammation in acute and chronic pancreatitis [58,59]. Therefore, we investigated whether TCT affected PARylation. In an enzyme activity assay, TCT inhibited PARP activity to a similar extent as the reference compound, 3-aminobenzamide (3-AB) (Figure 3A). The EC50 for 3-AB was 25 µM, and the EC50 of TCT was 18 µM.

Moreover, hydrogen peroxide-induced PAR synthesis in isolated acinar cells was abolished by 30 µM TCT, as indicated by the lack of PAR polymer formation (detected by IF and Western blotting; Figure 3B–E). Of note, a smear-like appearance of PARylated proteins as seen in Figure 3D is normal because acceptor proteins can be modified with a varying number of negatively charged ADP-ribose units, greatly affecting their electrophoretic mobility.

### 3.6. Tricetin Pretreatment Protects Mice from Cerulein-Induced Acute Pancreatitis

**Figure 4:**
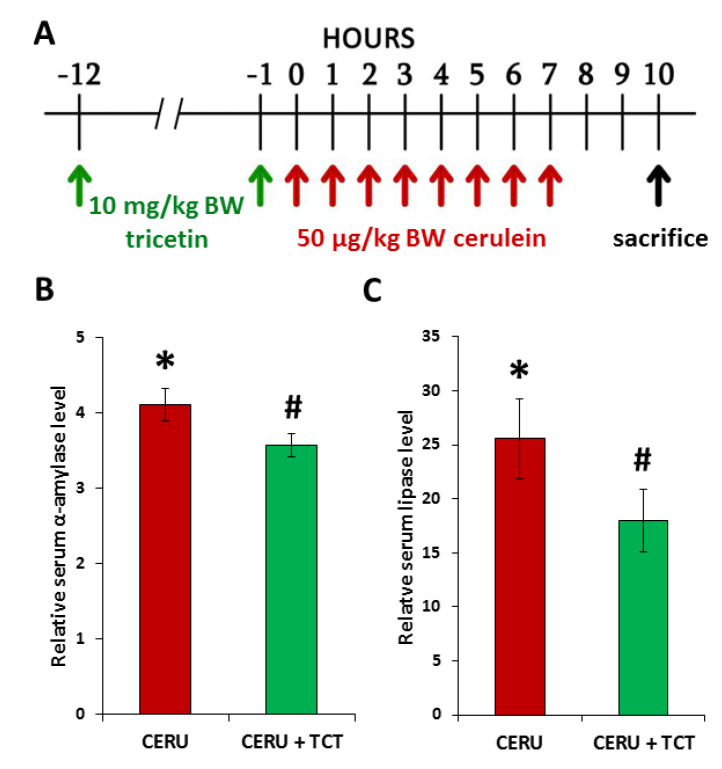
Tricetin inhibits tissue injury in acute pancreatitis. Acute pancreatitis was induced in mice with intraperitoneal injections (8 × 50 µg/kg BW) of cerulein at hourly intervals. The animals were sacrificed 10 h after the first cerulein injection. Tricetin (TCT) was applied as a pretreatment (2 × 10 mg/kg BW) in i.p. injections, 12 h and 1 h before the first cerulein injection (**A**). Serum levels of amylase and lipase were measured. Error bars represent SD of six mice. Statistical analysis was performed with one-way ANOVA test followed by Tukey’s post hoc test. The cerulein treatment caused significantly (* *p* < 0.05) elevated serum alpha amylase (**B**) and lipase (**C**) levels, which were significantly (# *p* < 0.05) reduced by TCT pretreatment (**B**,**C**).

Acute pancreatitis was induced in mice with cerulein, and mice were terminated 3 h after the last cerulein injection (Figure 4A).

**Figure 5:**
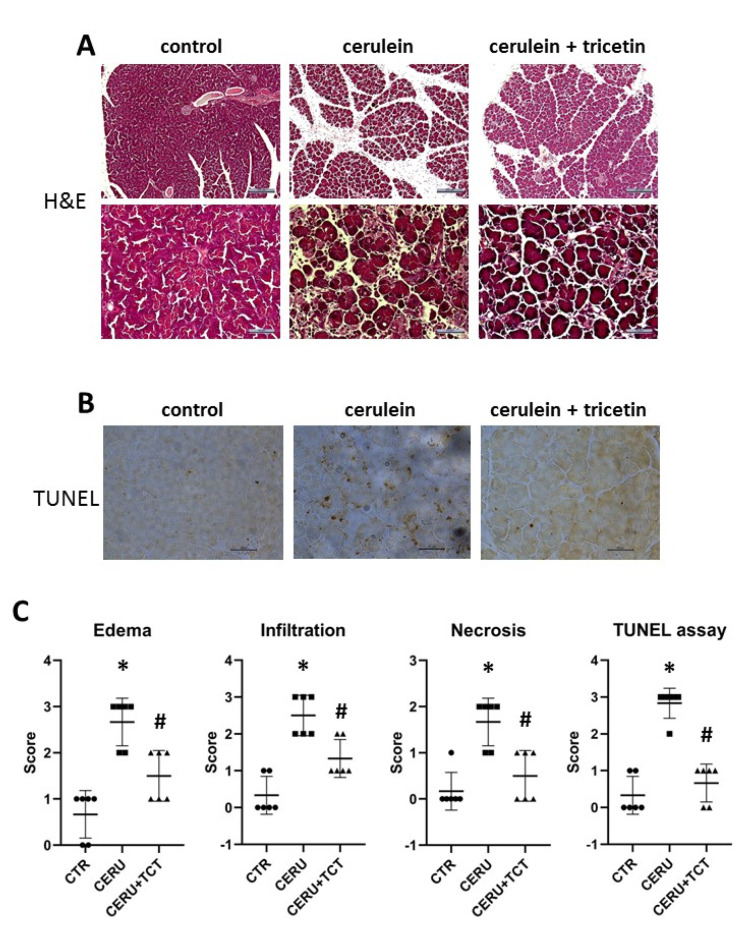
Tricetin inhibits tissue injury, edema, apoptosis, and necrosis in acute pancreatitis. Micrographs of hematoxylin and eosin-stained pancreas sections from mice treated with saline, cerulein, or cerulein + tricetin (TCT) (scale bar in the top row is 200 μm and in the bottom row is 50 μm) (**A**). Micrographs of pancreas sections stained for TUNEL assay from the same three groups of mice (scale bar is 50 μm) (**B**). Edema, leukocyte infiltration, apoptosis (TUNEL assay), and necrosis scores (0–3) were determined by a pathologist (**C**). Error bars represent SD of six mice. Statistical analysis was performed with Fisher’s exact test. Stars (*) indicate significant (*p* < 0.05) differences between cerulein-treated and control groups. Hashtags (#) indicate significantly (*p* < 0.05) reduced scores in the TCT group versus the cerulein-treated group.

The effects of TCT (10 mg/kg BW) given at 1 and 12 h before the first cerulein injection were examined. The TCT dose was selected from in vivo studies in which a similar compound fisetin (a flavonol) was investigated [37]. As expected, cerulein caused acinar cell injury indicated by the elevated serum levels of lipase and amylase (Figure 4B). TCT significantly reduced acinar cell injury, as reflected by decreased serum lipase and amylase levels (Figure 4B). Moreover, analysis of H&E-stained sections revealed edema in the pancreas of cerulein-treated mice (Figure 5A). TCT prevented edema formation, neutrophil infiltration, necrosis (Figure 5A), and formation of TUNEL positive cells/apoptotic bodies (Figure 5B), as verified by semiquantitative analysis of the sections (Figure 5C).

### 3.7. Tricetin Suppresses Granulocyte Infiltration, Production of Inflammatory Mediators, and Synthesis of Poly(ADP-ribose) in Acute Pancreatitis

**Figure 6:**
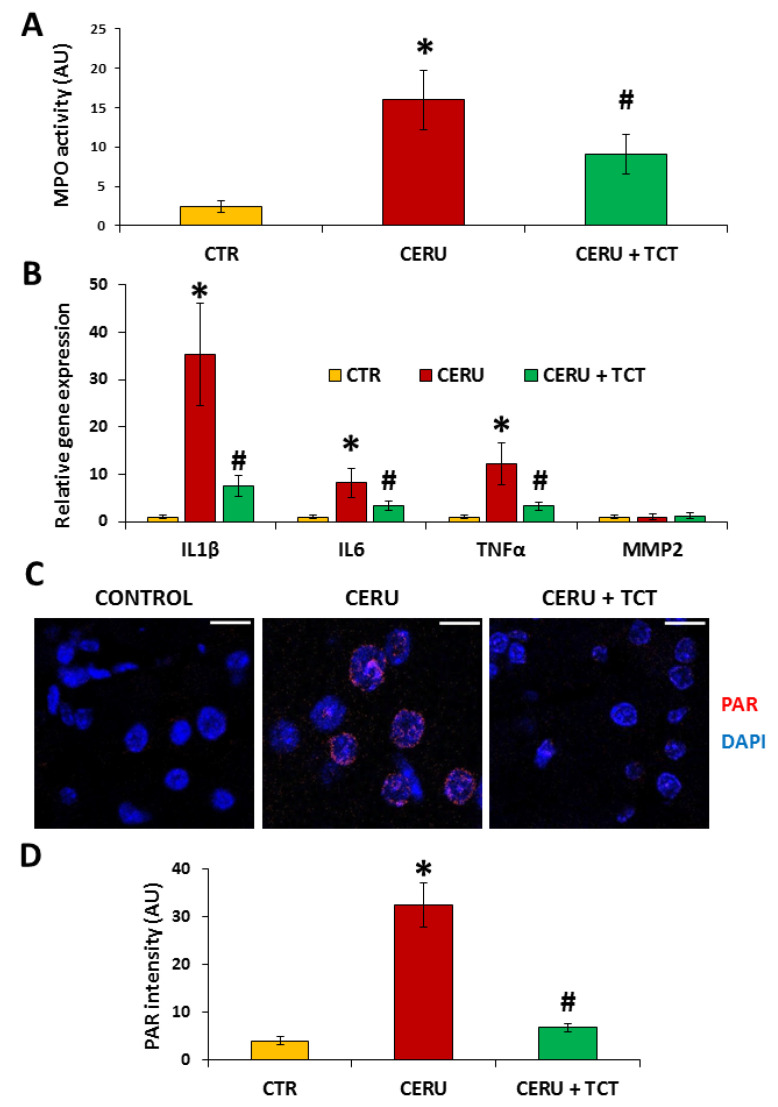
Tricetin inhibits inflammatory cell migration, PAR synthesis, and expression of inflammatory mediators in acute pancreatitis. Tricetin (TCT) treatment (2 × 10 mg/kg BW i.p.) suppressed neutrophil infiltration in the pancreas, as indicated by the decreased MPO level in the cerulein-induced acute pancreatitis model (**A**). TCT was effective in reducing IL1β, IL6, and TNFα expression, as indicated by the RT-qPCR assay data (**B**). Representative pictures of PAR immunofluorescent staining of pancreas sections are shown (**C**). The extent of PARylation was quantified by ImageJ software (**D**). TCT treatment (2 × 10 mg/kg BW i.p.) decreased PARylation in the cerulein-induced acute pancreatitis model (scale bar 20 μm) (**C**,**D**). Error bars represent the SD of six mice. Statistical analysis was performed with one-way ANOVA test followed by Tukey’s post hoc test. Stars (*) indicate significant (*p* < 0.05) differences between cerulein-treated and control groups. Hashtags (#) indicate significantly (*p* < 0.05) reduced scores in the TCT group versus the cerulein-treated group.

Tissue infiltration by white blood cells, especially granulocytes, is a hallmark of inflammation. Measuring tissue MPO levels is a good indicator of the granulocyte content of tissues. MPO activity increased close to sevenfold after induction of AP (Figure 6A). TCT treatment significantly reduced tissue MPO levels, suggesting suppression of granulocyte migration into the pancreas (Figure 6A).

The expression of IL1β and IL6 was upregulated in the pancreas in vivo (Figure 6B), similar to cerulein-treated isolated acinar cells. Pretreatment with TCT prevented the upregulation of IL1β and IL6 (Figure 6B). In contrast to the results in acinar cells, MMP2 levels did not change in vivo in the AP group and TCT did not affect MMP2 mRNA expression levels (Figure 6B). TNFα, an important mediator of AP in vivo [60] was not induced by cerulein in isolated acinar cells (Figure 2A). However, TNFα mRNA levels increased in the pancreas after cerulein treatment, and TCT treatment significantly inhibited TNFα expression (Figure 6B).

PARP1 is a central mediator of inflammation and programmed necrotic cell death. We detected poly(ADP-ribose) (PAR) polymer, the product of PARP1, in tissue sections. A high number of nuclei in the exocrine pancreas were immunopositive for PAR (Figure 6C), indicating acinar PARP activation in AP. In contrast, PAR polymers were barely detectable in pancreas sections from TCT-treated animals (Figure 6C,D).

### 3.8. Posttreatment with Tricetin Protects Mice from Cerulein-Induced Acute Pancreatitis

**Figure 7:**
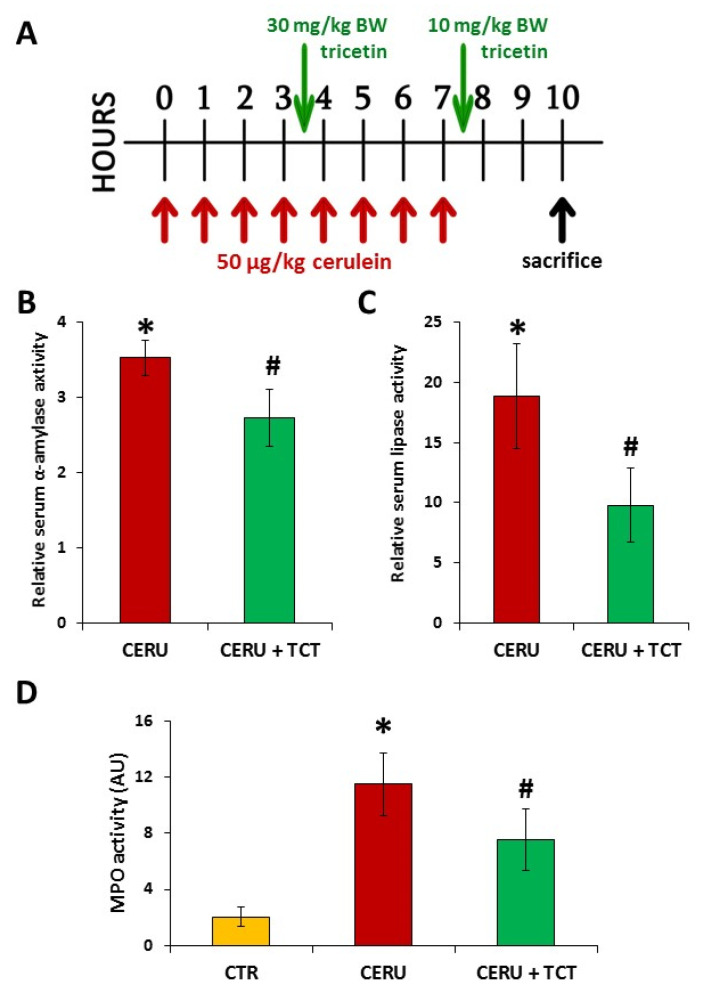
Posttreatment with tricetin inhibits tissue injury and inflammatory cell migration in acute pancreatitis. Acute pancreatitis was induced in mice with (8 × 50 µg/kg BW, i.p.) cerulein treatment at hourly intervals. The animals were sacrificed 10 h after the first cerulein injection. Tricetin (TCT) was administered between the 4th and 5th cerulein injections (30 mg/kg BW, i.p.) and after the last cerulein injection (10 mg/kg BW) (**A**). Serum levels of amylase and lipase were measured. Cerulein treatment significantly elevated serum α-amylase and lipase levels. This elevation in serum α-amylase and lipase levels was attenuated by TCT posttreatment (**B**,**C**). TCT suppressed neutrophil infiltration in the pancreas as indicated by the decreased tissue MPO levels (**D**). Error bars represent SD of six mice. Statistical analysis was performed with one-way ANOVA test followed by Tukey’s post hoc test. Stars (*) indicate significant (*p* < 0.05) differences between cerulein-treated and control groups. Hashtags (#) indicate significantly (*p* < 0.05) reduced values in the TCT group versus the cerulein-treated group.

The effects of TCT posttreatment on AP were examined (Figure 7A). TCT posttreatment significantly reduced acinar cell injury as reflected by decreased serum amylase and lipase levels (Figure 7B,C). Moreover, TCT significantly reduced pancreatic MPO levels, indicating suppression of granulocyte migration into the tissue (Figure 7D).

## 4. Discussion

Despite intensive preclinical and clinical research, we still do not have an effective therapy for acute pancreatitis. While several flavonoids mitigate AP, the flavone compound TCT has not yet been studied. Our results showed that TCT prevented acinar cell injury and death and inflammation in both cell and animal models of AP.

To investigate the effects of TCT in AP, we chose cerulein exposure as a commonly used model displaying key events of AP both in primary acinar cell culture and in vivo. In supraphysiologic concentrations, this cholecystokinin (CCK) analog peptide stimulates both CCK1 and CCK2 receptors. These G protein-coupled receptors trigger calcium signaling via PLC and inositol trisphosphate, resulting in reactive oxygen species (ROS) production, immune-inflammatory signaling (Jak/STAT, NFκB), and cell death [61]. Acinar cells also communicate with tissue macrophages in vivo to contribute to the pathogenesis of AP by inflammatory cytokine and chemokine production [62].

Cerulein triggered cell death in isolated primary murine acinar cells. Loss of cell viability in cerulein-treated acinar cells, as assessed by calcein assays, confirmed the toxic effects of CCK receptor overstimulation by cerulein. Moreover, cerulein-induced cell death had features of both apoptosis and necrosis, as indicated by caspase activation (apoptosis) as well as LDH release and propidium iodide uptake (indicators of necrosis). The significant cytoprotective effect of TCT could be observed both in the general viability assay as well as in apoptosis and necrosis assays. Moreover, TCT treatment helped maintain cellular viability in the mouse model of AP, as indicated by reduced serum amylase and lipase levels in TCT-treated animals compared with the serum levels in cerulein-treated mice. Interestingly, the effects of TCT were more pronounced on serum lipase activity than on amylase activity. This may be due to a more robust lipase release response compared to the less marked amylase release in the cerulein-treated animals. A previous study found that lipase is indeed a more sensitive indicator of pancreatitis [63]. Nevertheless, both serum markers confirmed the in vivo cytoprotective effects of TCT. TUNEL staining performed on pancreatic tissue sections also suggested that TCT prevents apoptotic cell death in vivo. The preserved pancreatic tissue architecture of TCT-treated animals also indicated less severe tissue injury in response to cerulein.

Since ROS production was implicated in cerulein-induced acinar cell injury and AP [64,65], we hypothesized that TCT may act, at least in part, by inhibiting ROS-induced cell death mediated by PARP1. Of note, the oxidative stress-induced and PARP1-mediated cell death pathway displays features of necrosis (including membrane permeabilization) [66], which are in line with the primarily necrotic nature of acinar cells in AP. Furthermore, some flavonoids have previously been shown to exert PARP inhibitory effects [67]. TCT inhibited PARP with a potency similar to 3-aminobenzamide. Moreover, in oxidatively-stressed primary pancreatic acinar cells, PAR polymer formation was observed, indicating PARP activation. TCT abolished PAR synthesis, as demonstrated in both immunofluorescent staining and Western blotting (Figure 3). Furthermore, in the cerulein-induced AP model, acinar cell nuclei contained PAR polymers, and TCT treatment abolished PAR formation (Figure 6). PARP1 and PARylation have been shown to contribute to the pathogenesis of AP [18,59]. Our current findings confirm that PARP inhibition by TCT translates to in vivo conditions, suggesting that PARP inhibition may be a key factor in the protection provided by TCT in AP. The cytoprotective effects of flavonoids might also be due to their effects on Akt signaling [68].

Oxidative stress is a common feature of various forms of inflammation, and it also plays a key role in the pathomechanism of AP. We hypothesized that an antioxidant mechanism may contribute to the protective effects of TCT in AP. Indeed, TCT had radical scavenging activity, as shown in the ABTS free radical capture assay. Interestingly, however, TCT did not protect against hydrogen peroxide-induced acinar cell injury (Supplementary Figure S2B–D). This may be due to the lack of hydrogen peroxide-specific antioxidant activity at the TCT concentration used in the assay. Moreover, instead of scavenging free radicals, antioxidants are known to act primarily by increasing the nucleophilic tone [69], inducing an antioxidant response via Nrf2 activation. Whether or not this is the case with TCT, requires further investigation.

Our data clearly indicate that TCT suppresses inflammation in AP. Reduced edema, suppressed inflammatory cell migration, and preserved tissue architecture support this statement (Figure 5). The underlying mechanism for the anti-inflammatory effects of TCT involves inhibition of the production of inflammatory mediators. From the cell-based experiments, we established that TCT inhibits the activation of inflammatory mediator genes (Figure 2). Our in vivo data extended this list with TNFα as a central mediator of inflammation induced by cerulein [70] and suppressed by TCT (Figure 6). TNFα was not induced in acinar cell-based experiments, in line with our current understanding that macrophages are the main source of this cytokine in AP [71]. Thus, TCT may suppress inflammatory (cytokine, chemokine, MMP) gene activation both in acinar cells and in macrophages. We hypothesized that inhibition of NFκB may be the underlying mechanism for this effect. Indeed, our data proved that TCT inhibits NFκB activation in acinar cells (Figure 2B). It had no direct effect on the binding of the transcription factor to its consensus sequence (Figure 2C), suggesting that TCT interferes with a proximal step in the NFκB signaling pathway. The effect of TCT on NFκB may also be due to the PARP inhibitory effect of the drug, as PARP1 acts as a coactivator of NFκB and PARP inhibition/PARP1 knockout suppresses NFκB in a wide range of settings [58,72,73].

Although damage to acinar cells is the most studied cause of pancreatitis and the focus of our studies is also on these cells, we cannot ignore the fact that the disease is not only due to the failure of these cells. Early protease activation and NFκB activation are essential features of AP; both occur in parallel during disease manifestation and strongly influence each other. However, not only protease and NFκB activation play a critical role, but also the cell type in which their activation occurs is important [74].

Although the focus of this work was on the antioxidant and PARP inhibitory effect of TCT, it must be noted that TCT may also have additional effects contributing to its protection against pancreatitis. Pancreatic ischemia plays a dominant role in the development of AP, as well as in the progression of the disease into severe forms [75,76,77]. This statement is supported by observations that reduction in pancreatic blood flow increases the severity of acute pancreatitis [78], whereas improvement of pancreatic blood flow reduces the severity of acute pancreatitis and accelerates pancreatic recovery in this disease [79,80]. Previous studies have shown that the flavonoid quercetin [81,82] increased pancreatic blood flow in AP which translated into tissue protective effects and accelerated pancreatic recovery. Such observations suggest that the protective effect of tricetin on the pancreas in cerulein-induced AP may be, at least in part, related to the improvement of pancreatic blood flow.

## 5. Conclusions

In summary, TCT can be a novel candidate for the treatment of AP. TCT inhibits two central pathways of AP pathophysiology: acinar cell death and inflammation. The antioxidant and PARP inhibitory effects of TCT may play a central role in the protective effect of this flavone compound in AP.

## Figures and Tables

**Table 1:** Primers used for RT-PCR.

Gene	Forward Primers (5→3)	Reverse Primers (5→3)
IL1β	CAACCAACAAGTGATATTCTCCATG	GATCCACACTCTCCAGCTGCA
IL6	GAGGATACCACTCCCAACAGACC	AAGTGCATCATCGTTGTTCATACA
IL10	GGCGCTGTCATCGATTTCTC	ATGGCCTTGTAGACACTTTGG
TNFα	CATCTTCTCAAAATTCGAGTGACAA	TGGGAGTAGACAAGGTACAACCC
TGFβ	CCGCAACAACGCCATCTATG	GTTCCACATGTTGCTCCACAC
IFNγ	GGAACTGGCAAAAGGATGGTG	ATGTTGTTGCTGATGGCCTG
CCL5	TGCAGTCGTGTTTGTCACTC	AGAGCAAGCAATGACAGGGA
CXCL10	GATGACGGGCCAGTGAGAAT	CGTGGCAATGATCTCAACAC
MMP2	AACGGTCGGGAATACAGCAG	GTAAACAACGCTTCATGGGGG
36B4	GGACCCGAGAAGACCTCCTT	GCACATCACTCAGAATTCAATCC
RPS26	CCACAATTCAGACCTGCTG	GGGTAATTTTCCTTCCGTCCT

**Table 2:** Histologic scoring of tissue sections.

	0	1	2	3
Edema	no edema	interlobular edema	interlobular and moderate intralobular edema	interlobular and severe intralobular edema
Infiltration	absent	low perivascular infiltration	moderate perivascular and low diffuse infiltration	severe diffuse infiltration
Necrosis	none or negligible	observed in one third of the cells	observed in half of the cells	observed in most cells
TUNEL	0–5 average spot number/400× field	6–15 average spot number/400× field	16–35 average spot number/400× field	>5 average spot number/400× field

## Data Availability

Data supporting reported results have been deposited in Zenodo (doi:10.5281/zenodo.6526470).

## Supplementary Materials

The following supporting information can be downloaded at: https://www.mdpi.com/article/10.3390/biomedicines10061371/s1, Figure S1: Low concentrations (1–30 μM) of tricetin are nontoxic to acinar cells; Figure S2: Tricetin did not affect hydrogen peroxide-induced acinar cell death.
