# Supplementary material for: Tricetin Reduces Inflammation and Acinar Cell Injury in Cerulein-Induced Acute Pancreatitis: The Role of Oxidative Stress-Induced DNA Damage Signaling
Source: Biomedicines. 2022 Jun 10;10(6):1371. doi: 10.3390/biomedicines10061371 (PMC9219693; doi:10.3390/biomedicines10061371)
Supplement: Supplementary file 1 [file biomedicines-10-01371-s001.zip › biomedicines-1738398-supplementary.pdf]

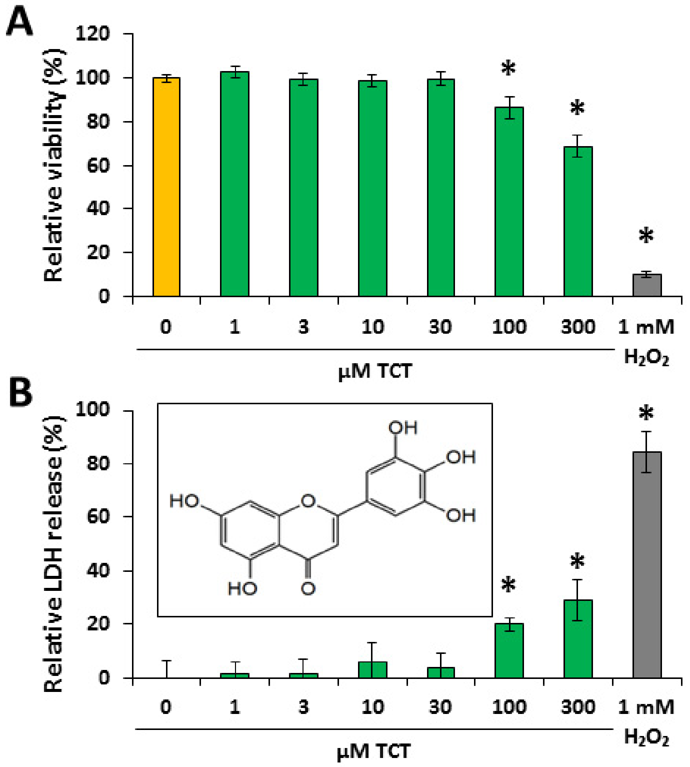

Figure S1. Up to a concentration of 30 µM, tricetin (TCT) is not toxic to isolated acinar cells. Primary acinar cells were isolated from mice and were used for testing the toxicity of TCT. Cell viability was evaluated after 24 h of TCT treatement with calcein assay (**A**) and LDH release assay (**B**) using hydrogen peroxide (1 mM) as a positive control. We found that TCT was non-toxic to primary acinar cells up to a concentration of 30 µM. At three times higher concentration (100 µM), TCT had a small but significant toxic effect on acinar cells. This could be observed in both the calcein and LDH release assays (**A,B**). *, $p < 0.05$.

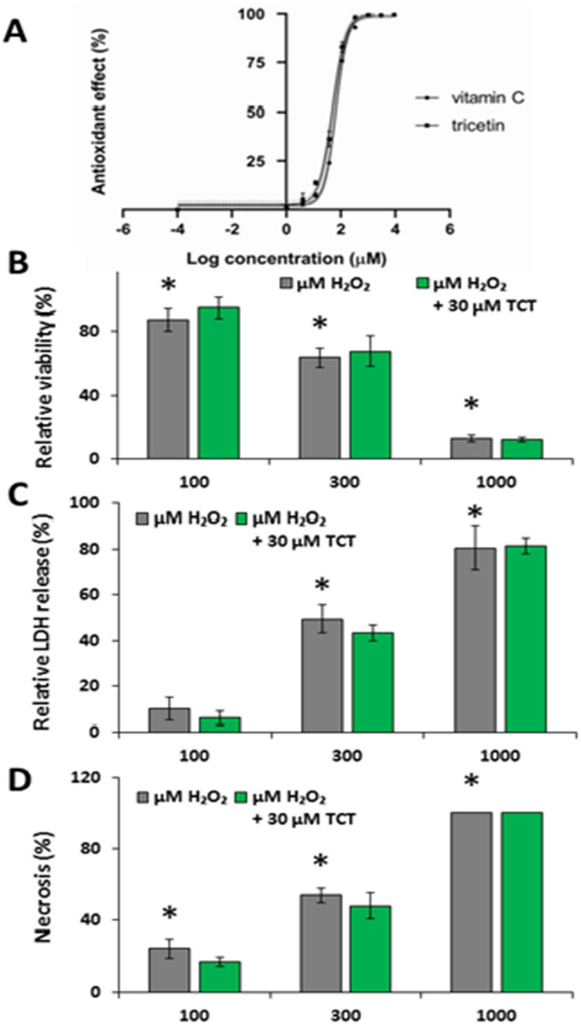

Figure S2. Tricetin is an antioxidant but has no effect of hydrogen peroxide-induced cytotoxicity. The antioxidant activity of tricetin (TCT) was determined with the ABTS radical scavenging assay using vitamin C as positive control (A). TCT has an antioxidant effect similar to vitamin C (A). The IC50 for TCT was 50 µM and the IC50 for vitamin C was 65 µM (A). Primary acinar cells were pretreated with 30 µM TCT for 1 hour and then treated with the indicated concentrations of hydrogen peroxide for 24 hours. Viability was then determined with the calcein assay (B) and necrotic cell death was measured with LDH release assay (C) and propidium iodide (PI) uptake assays (D). TCT did not significantly change H_2O_2-induced cytotoxicity. Error bars represent the SD of three independent experiments. Stars (*) indicate significant (p<0.05) hydrogen peroxide-induced cell death versus control.
